# Participation in a Hospital Incentive Program for Follow-up Treatment for Opioid Use Disorder

**DOI:** 10.1001/jamanetworkopen.2019.18511

**Published:** 2020-01-03

**Authors:** Austin S. Kilaru, Jeanmarie Perrone, David Kelley, Sari Siegel, Su Fen Lubitz, Nandita Mitra, Zachary F. Meisel

**Affiliations:** 1 National Clinician Scholars Program, University of Pennsylvania, Philadelphia; 2 Corporal Michael J. Crescenz VA Medical Center, Philadelphia, Pennsylvania; 3 Department of Emergency Medicine, Perelman School of Medicine, University of Pennsylvania, Philadelphia; 4 Penn Injury Science Center, University of Pennsylvania, Philadelphia; 5 Office of Medical Assistance Programs, Department of Human Services, Commonwealth of Pennsylvania, Harrisburg; 6 The Hospital and Healthsystem Association of Pennsylvania, Harrisburg; 7 Department of Biostatistics and Epidemiology, Perelman School of Medicine, University of Pennsylvania, Philadelphia

## Abstract

This cross-sectional study examines hospital participation in an incentive program that aims to improve the rate of opioid use disorder follow-up treatment among Medicaid recipients in Pennsylvania.

## Introduction

Pennsylvania experienced an 80% increase in emergency department (ED) visits for opioid overdose from 2016 to 2017.^1^ The engagement of patients with opioid use disorder (OUD) in treatment after hospital discharge is a key strategy in preventing subsequent opioid overdose.^2,3^ The Pennsylvania Department of Human Services established an incentive program to improve the rate of OUD follow-up treatment among Medicaid recipients.^4^ In the Opioid Hospital Quality Improvement Program, hospitals earned payment for designing and attesting to 4 distinct clinical pathways: (1) ED initiation of buprenorphine treatment, (2) warm handoff to community resources, (3) referral and treatment for pregnant patients, and (4) inpatient initiation of medication treatment. Payment of the full incentive ($193 000) was contingent on participation and attestation of all 4 pathways, with smaller incentives for partial participation. We evaluated participation in this program among hospitals.

## Methods

This study was deemed to be exempt from review by the institutional review board at the University of Pennsylvania. Because this study was done with publicly reported data, no informed consent was required by the institutional review board. We conducted a cross-sectional analysis of all hospitals with an ED in Pennsylvania. We excluded pediatric, federal, and specialty hospitals. Participation in the program was publicly reported in January 2019.^4^ We obtained publicly reported data on hospital characteristics from the Pennsylvania Department of Health and county-level data from the Pennsylvania Open Data Portal.^5,6^ We used a multivariable logistic regression model with robust SEs to compare differences in characteristics of hospitals that fully participated with those that declined or partially participated. We report adjusted risk differences (ARDs) and corresponding 95% CIs. A 2-sided *P* < .05 was deemed to be statistically significant. Analyses were conducted using Stata, version 14 (StataCorp LLC). This study followed the Strengthening the Reporting of Observational Studies in Epidemiology (STROBE) reporting guideline.

## Results

Of 155 hospitals that met the inclusion criteria, 79 (51%) attested to all 4 pathways, 45 (29%) attested to fewer than 4 pathways, and 31 (20%) declined to attest to any pathway (Table 1). A total of 93 hospitals (60%) attested to the first pathway, 124 (80%) to the second pathway, 118 (76%) to the third pathway, and 93 (60%) to the fourth pathway. Fully participating hospitals had a mean (SD) bed size of 250 (242), and partial or nonparticipating hospitals had a mean (SD) bed size of 163 (125). In the adjusted model, larger hospitals were full participants more often than smaller hospitals (ARD, 5 percentage points; 95% CI, 0.2-10 percentage points; *P* = .04) (Table 2). Hospitals affiliated with health systems were full participants more often than independent hospitals (ARD, 22 percentage points; 95% CI, 4-42 percentage points; *P* = .02). Compared with hospitals in the southeast region of the state, hospitals in the northeast region were full participants less often (ARD, −34 percentage points; 95% CI, −56 to −12 percentage points; *P* = .01), and hospitals in the central and western regions were full participants more often (central: ARD, 27 percentage points; 95% CI, 12-43 percentage points; *P* = .004; western: ARD, 38 percentage points; 95% CI, 22-53 percentage points; *P* < .001). Hospitals in counties with higher overdose rates were more often full participants (ARD, 5 percentage points; 95% CI, 2-8 percentage points; *P* = .006).

**Table 1.** Characteristics of Pennsylvania Hospitals by Level of Participation in Opioid Hospital Quality Improvement Program

Characteristic	Hospitals^a^			
	All (N = 155)	Fully Participating (n = 79)	Partially Participating (n = 45)	Nonparticipating (n = 31)
Bed size, mean (SD), No.	208 (198)	250 (242)	187 (142)	129 (86)
Location, No. (%)				
Urban	114 (74)	59 (75)	36 (80)	19 (61)
Rural	41 (26)	20 (25)	9 (20)	12 (39)
Teaching status, No. (%)				
Teaching	86 (55)	49 (62)	23 (51)	14 (45)
Nonteaching	69 (45)	30 (38)	22 (49)	17 (55)
Tax status				
Not for profit	145 (94)	74 (94)	45 (100)	26 (84)
For profit	10 (6)	5 (6)	0	5 (16)
Health system affiliation, No. (%)				
Independent	28 (18)	9 (11)	9 (20)	10 (32)
≥2 Hospitals	127 (82)	70 (89)	36 (80)	21 (68)
State region, No. (%)				
Southeastern	47 (30)	21 (26)	18 (40)	8 (26)
Northeastern	22 (14)	3 (4)	9 (20)	10 (32)
Central	33 (21)	19 (24)	11 (24)	3 (10)
Western	53 (34)	36 (46)	7 (16)	10 (32)
State-designated Opioid Center of Excellence located within county, No. (%)^b^	105 (68)	58 (73)	29 (64)	18 (58)
Licensed OUD treatment facilities in county, mean (SD), No.	32 (36)	42 (42)	24 (24)	19 (25)
Rate of overdose ED visits in 2018, mean (SD), No./10 000 county residents	9.6 (3.9)	10.3 (4.4)	9.0 (3.3)	8.4 (3.3)
Dispensed prescriptions of buprenorphine in 2018, mean (SD), No./1000 county residents	25.3 (15.4)	26.5 (14.3)	23.1 (16.1)	25.5 (17.4)
County residents living in poverty in 2018, mean (SD), %	12.8 (5.1)	14.3 (5.3)	10.8 (4.6)	12.2 (3.9)

Abbreviations: ED, emergency department; OUD, opioid use disorder.

^a^ Fully participating hospitals attested to all 4 treatment pathways, partially participating hospitals attested to fewer than 4 pathways, and nonparticipating hospitals did not attest to any treatment pathways.

^b^ Pennsylvania Centers of Excellence for Opioid Use Disorder are state-designated specialized treatment centers that provide comprehensive care for patients with OUD.

**Table 2.** Differences in Characteristics Between 79 Fully Participating Hospitals and 76 Partially and Nonparticipating Hospitals

Characteristic	Adjusted Risk Difference, % (95% CI)^a^	Odds Ratio (95% CI)	*P* Value
Bed size, actual No. ^b^	5 (0.2 to 10)	1.4 (1.0 to 1.9)	.04
Urban location	−2 (−17 to 20)	0.9 (0.3 to 2.7)	.87
Teaching hospital	−3 (−20 to 13)	0.8 (0.3 to 2.2)	.71
Independent hospital	−22 (−42 to −4)	0.3 (0.09 to 0.9)	.02
State region			
Southeastern	1 [Reference]	NA	NA
Northeastern	−34 (−56 to −12)	0.1 (0.03 to 0.7)	.01
Central	27 (12 to 43)	5.7 (1.8 to 18.0)	.004
Western	38 (22 to 53)	9.4 (3.1 to 28.8)	<.001
Rate of overdose ED visits in Q3 2018, No./10 000 county residents^b^	5 (2 to 8)	1.2 (1.1 to 1.4)	.006
Opioid Center of Excellence located within county	5 (−12 to 21)	1.3 (0.5 to 3.5)	.59

Abbreviations: ED, emergency department; NA, not applicable; Q3, third quarter.

^a^ Differences in outcomes were estimated using a multivariable logistic regression model with robust SEs. A multivariable logistic regression model that accounted for correlated data by health system using generalized estimating equations with an independent correlation structure with robust SEs demonstrated identical odds ratios and equivalent statistical significance.

^b^ For continuous covariates, the adjusted risk difference shown in this table is the difference between the predicted probability of the outcome at the mean value of the covariate for fully participating hospitals and the predicted probability at the mean value for partial and nonparticipating hospitals.

## Discussion

Pennsylvania introduced the first statewide financial incentive to engage patients with OUD in treatment after hospital discharge. Although most hospitals participated in the program, more were willing to arrange warm handoffs to community treatment facilities rather than initiate medication treatment for OUD. Study limitations include the focus on pathway adoption rather than patient outcomes and not accounting for partnerships between hospitals and community treatment resources. In future years of the program, hospitals can earn payments for annual improvement in the rate of OUD follow-up treatment. Policies seeking to facilitate this transition among all hospitals and communities should consider local barriers to treatment initiation and follow-up.
